# Understanding structured medication reviews delivered by clinical pharmacists in primary care in England: a national cross-sectional survey

**DOI:** 10.1136/bmjopen-2024-097012

**Published:** 2025-09-30

**Authors:** Adaku J Agwunobi, Anna E Seeley, Katherine L Tucker, Paul A Bateman, Christopher Elles Clark, Andrew Clegg, Gary Ford, Seema Gadhia, F D Richard Hobbs, Kamlesh Khunti, Gregory Y H Lip, Simon de Lusignan, Jonathan Mant, Deborah McCahon, Rupert A Payne, Rafael Perera, Samuel Seidu, James P Sheppard, Marney Williams, Cynthia Wright-Drakesmith, Richard J McManus, Rebecca K Barnes

**Affiliations:** 1 Nuffield Department of Primary Care Health Sciences, University of Oxford, Oxford, UK; 2 Exeter Collaboration for Academic Primary Care, University of Exeter Medical School, Exeter, UK; 3 University of Leeds School of Medicine, Leeds, West Yorkshire, UK; 4 Bradford Teaching Hospitals NHS Foundation Trust, Bradford, Bradford, UK; 5 Radcliffe Department of Medicine, University of Oxford, Oxford, UK; 6 Oxford and Thames Valley, Health Innovation Network, Oxford, London, UK; 7 University of Leicester Diabetes Research Centre, Leicester, UK; 8 Liverpool Centre for Cardiovascular Science, University of Liverpool, Liverpool, UK; 9 Department of Clinical Medicine, Aalborg Universitet, Aalborg, Region Nordjylland, Denmark; 10 University of Cambridge Primary Care Unit, Cambridge, UK; 11 Population Health Sciences, Bristol Medical School, Bristol, UK; 12 University of Leicester, Leicester, UK; 13 Brighton and Sussex Medical School, Brighton, UK

**Keywords:** Polypharmacy, Pharmacists, Primary Health Care, Medication Review, Surveys and Questionnaires

## Abstract

**Abstract:**

**Objectives:**

This study explored how Structured Medication Reviews (SMRs) are being undertaken and the challenges to their successful implementation and sustainability.

**Design:**

A cross-sectional mixed methods online survey.

**Setting:**

Primary care in England.

**Participants:**

120 clinical pharmacists with experience in conducting SMRs in primary care.

**Results:**

Survey responses were received from clinical pharmacists working in 15 different regions. The majority were independent prescribers (62%, n=74), and most were employed by Primary Care Networks (65%, n=78), delivering SMRs for one or more general practices. 61% (n=73) had completed, or were currently enrolled in, the approved training pathway. Patient selection was largely driven by the primary care contract specification: care home residents, patients with polypharmacy, patients on medicines commonly associated with medication errors, patients with severe frailty and/or patients using potentially addictive pain management medication. Only 26% (n=36) of respondents reported providing patients with information in advance. The majority of SMRs were undertaken remotely by telephone and were 21–30 min in length. Much variation was reported in approaches to conducting SMRs, with SMRs in care homes being deemed the most challenging due to additional complexities involved. Challenges included not having sufficient time to prepare adequately, address complex polypharmacy and complete follow-up work generated by SMRs, issues relating to organisational support, competing national priorities and lack of ‘buy-in’ from some patients and General Practitioners.

**Conclusions:**

These results offer insights into the role being played by the clinical pharmacy workforce in a new country-wide initiative to improve the quality and safety of care for patients taking multiple medicines. Better patient preparation and trust, alongside continuing professional development, more support and oversight for clinical pharmacists conducting SMRs, could lead to more efficient medication reviews. However, a formal evaluation of the potential of SMRs to optimise safe medicines use for patients in England is now warranted.

**STRENGTHS AND LIMITATIONS OF THIS STUDY**

- This cross-sectional mixed methods survey study is the first to describe how Structured Medication Reviews (have been implemented at scale by clinical pharmacists working in primary care settings across England.
- NHS England stakeholders, pharmacy professionals, pharmacy educators and patient representatives were consulted throughout to ensure relevant and appropriate questions were asked in the survey, and to advise on the survey distribution strategy.
- The survey consisted of both closed-ended questions to enable speed of response and easily quantifiable data, and open-ended questions to enable more in-depth free-text responses.
- Our findings represent a ‘snap-shot’ of 120 clinical pharmacists’ experiences at a specific point in time. Patients and General Practitioners may have had a different view.
- Due to the methods of the distribution and the anonymous nature of the survey, it was not possible to report survey response metrics such as participation or completeness rates. The survey may have been subject to respondent bias by relying on third-party efforts at distribution and voluntary participation with no reimbursement.

## Introduction

 The long-term prescription of multiple medications to individuals, known as polypharmacy, increases with age such that more than one in 10 people aged over 65 years take at least eight different prescribed medications weekly, increasing to one in four among people who are aged over 85.^1^ At least 16% of hospital admissions are thought to be linked to adverse reactions to prescribed medication, particularly in individuals taking ten or more medicines and with six or more comorbidities.^2^ 237 million medication errors are estimated to occur annually across primary and secondary care settings in England, costing the National Health Service (£98.4 million each year.^3^ With an ageing population and increasing multiple long-term conditions (MLTC or multimorbidity), improving the appropriate use of polypharmacy and reducing medicines-related problems in older people has become a priority.^4^

One approach to meeting this challenge is to undertake explicit and systematic medication reviews, described by Shaw and Seal as a ‘structured, critical examination of a patient’s medicines’.^5^ Previously, the scope of medication reviews has varied, with different types of reviews undertaken by different healthcare professionals in different settings.^6^ In 2015, a National Institute for Health and Care Excellence (NICE) evidence review concluded that there was mixed evidence as to whether medication reviews were more clinically effective in reducing the suboptimal use of medicines and medicines-related patient safety incidents compared with usual care.^7^ NICE recommended that primary care organisations consider carrying out a structured medication review (SMR) for individuals taking multiple medicines and/or living with chronic or long-term conditions, and older people. Furthermore, the most appropriate healthcare professional to carry out such a review should be determined locally, based on their knowledge and skills, for example, a clinical pharmacist or an appropriate member of a multidisciplinary team.^7^ The term ‘clinical pharmacist’ is generally understood to mean pharmacists working with patients and other healthcare professionals in a healthcare setting, like a hospital or general practitioner (GP) practice, rather than a community pharmacy setting.

The main objective of SMRs was defined as: ‘reaching an agreement with the person about treatment, optimising the impact of medicines, minimising the number of medication-related problems and reducing waste’.^7^ SMRs were formally introduced via NHS England (NHSE) in September 2020,^8^ as distinct from the act of re-authorising repeat prescriptions or a review of specific medicines during a long-term condition review. However, full implementation was delayed due to the COVID-19 pandemic.^9^

In 2022–2023, NHSE provided financial incentivisation for SMRs conducted by clinical pharmacists for patients in the following priority cohorts: people living in care homes, with complex and problematic polypharmacy (specifically taking ten or more medications), taking medicines which are commonly associated with medication errors, living with severe frailty and using potentially addictive pain management medications.^10^ Further incentives were provided to review patients on specific medication combinations or with omissions viewed as harmful. For instance, people over the age of 65 years who had been given a non-steroidal anti-inflammatory drug without gastric protection.^11^ However, these further incentives were discontinued in 2023/24, without notice.

Earlier research has mostly examined the history behind SMRs or undertaken small-scale trials.^12^ Real-world data on the practical difficulties and successes that come with integrating a new workforce into primary care and rolling out SMRs across the country is lacking.^9^ A qualitative interview study with clinical pharmacists delivering SMRs remotely in primary care in England between 2020–2022 reported that early implementation, without the opportunity for adequate clinical knowledge development, consultation training provision and skills acquisition, fell short of policy aspirations for holistic, personalised reviews.^13 14^ Therefore, the aim of the current study was to gain insight into the implementation of SMRs in primary care in England 3 years after their introduction and within the context of NHSE policy-making.

## Methods

### Study design

This was a cross-sectional mixed methods survey conducted between February and May 2023. Prior to commencing, a study protocol was developed, and NHS ethical approval was gained from South Central—Hampshire A Research Ethics Committee (Ref. 22/SC/0373). Eligibility criteria for participation were pharmacists with experience in conducting SMRs with primary care patients in England. We did not seek consent from our participants as the survey was anonymous. On the introductory page of the survey, it stated, ‘By filling out the survey, you are giving your consent for your responses to be used in our study. All responses will be anonymous’. The survey was voluntary and consisted of 43 questions over seven pages: closed-ended questions to enable speed of response and easily quantifiable data, and open-ended questions to enable more in-depth free-text responses. There was an option for respondents to save their responses and complete them later. If participants wished to leave their name and contact details to register their interest for future research, these contact details were separated from their survey responses.

A request for distribution (via their Twitter accounts and primary care newsletters) of an invitation to clinical pharmacists delivering SMRs in primary care in England to complete an anonymous ‘open’ e-survey, with a weblink to information about the study, was sent to the 15 regional Academic Health Science Networks. The same request was also sent to the 15 regional National Institute for Health Research Applied Research Collaborations. Our distribution strategy was to try and reach Medicines Optimisation leads and Chief Pharmacists working at the locality level, providing medicines leadership for Integrated Care Boards who would forward the invitation to clinical pharmacists delivering SMRs in primary care in England via their own networks. We also targeted national pharmacy educators and pharmacy champions with the same request to distribute the weblink to their clinical pharmacy networks. The original target was 30–50 respondents.

### Development of materials and procedure

We planned to report on the attributes of our respondents (eg, their employment and prescribing status), their experiences conducting SMRs and their perceptions (eg, challenges faced). We were not aware of any existing surveys on SMRs at the time. Drawing on published SMR guidance, several drafts of the survey were co-developed with clinical study team members, two clinical pharmacists delivering SMRs in primary care settings, three senior pharmacists (including care home leads) and three pharmacy educators. The survey questions were carefully selected to align with the study’s aim of gaining insight into everyday practice and the work involved in conducting SMRs, as well as exploring the challenges faced. Our approach to data validation included field-testing to seek feedback on question topics, phrasing, formatting, response options, logic and time to complete before refining and launching the survey.

A final version of the survey was created and launched using Jisc Online Surveys V.2 on 10 February 2023 (online supplemental data 1). On the first page of the survey, participants were informed about the purpose of the study, the nature of the questions the expected completion time, and who the principal investigator and funder were. The second page contained an eligibility screening question. The survey was open for 3 months with no financial incentive for completion.

### Data analysis

Responses were captured automatically by the survey platform. Quantitative data from closed-ended questions were analysed descriptively using functionality within the Jisc Online Surveys package (ie, tools for basic data analysis and reporting, allowing users to visualise, filter and interpret results and to download the data for further analysis). Qualitative data from open-ended questions were downloaded in preparation for thematic analysis^15^ using NVivo V.12. NVivo is a software program that is commonly used to support thematic analysis of qualitative data. It allows researchers to organise, code and analyse data from various sources, including surveys, making the process more efficient and systematic. An inductive approach was used to generate themes from the open-ended responses. Four members of the research group met and worked independently through the responses to each of the open-ended questions line-by-line to develop an initial set of codes and categories. These codes and categories were discussed and agreed. The qualitative data were then imported into NVivo, and the agreed codes were systematically applied to the data.

### Stakeholder, patient and public involvement

NHSE stakeholders, pharmacy professionals, pharmacy educators and patient representatives were consulted throughout the study to ensure relevant and appropriate questions were asked in the survey, to advise on the survey distribution strategy, and how best to disseminate the findings. The preliminary and final survey results were presented to the study expert stakeholder group, who confirmed the importance of the findings. One co-author is a patient representative, was a co-applicant on the funding application, supported development of the study protocol and participated in all investigator team meetings.

## Results

### Participant characteristics

Respondents were automatically assigned a unique response number, which did not contain any identifiable information about them. Due to the methods of distribution and the anonymous nature of the survey, it was not possible to report survey response metrics such as participation or completeness rates.^16^ NHSE workforce data for 31 January 2023 reports 4102 full-time equivalent clinical pharmacists in 1198 primary care networks (PCN).^17^ However, because the number of clinical pharmacists conducting SMRs was not reported, it was not possible to calculate a response rate for eligible participants.

The survey was completed by 120 clinical pharmacists conducting SMRs across England, with the majority of responses coming from the West of England and West Midlands. The majority of respondents were employed on permanent contracts with PCNs (65%, n=78) or general practices (25%, n=30), with a minority employed by other organisations. Just over half (51%, n=62) were conducting SMRs for two or more general practices. A small number of respondents (10%, n=12) were remote workers. The majority (65%, n=78) reported 2 years or less experience in conducting SMRs. 11% (13/120) of respondents reported six or more years of experience in conducting SMRs, despite them being introduced for the first time in 2020 (see table 1).

**Table 1:** Respondent demographics

Demographic	Respondents (n (%))
**Region**	
East of England	9 (7.6%)
East Midlands	8 (6.7%)
Greater Manchester	6 (5%)
Kent, Surrey and Sussex	3 (2.5%)
North East and North Cumbria	4 (3.4%)
North Thames	3 (2.5%)
North West London	6 (5%)
North West Coast	9 (7.6%)
Oxford and Thames Valley	8 (6.7%)
South London	10 (8.4%)
South West Peninsula	8 (6.7%)
Wessex	10 (8.4%)
West	16 (13.4%)
West Midlands	15 (12.6%)
Yorkshire and Humber	5 (4.2%)
**Length of time delivering SMRs**	
0–5 months	13 (10.8%)
6–11 months	14 (11.7%)
1–2 years	51 (42.5%)
3–5 years	29 (24.2%)
6 or more years	13 (10.8%)
**Training and background**	
Pharmacist in general practice pathway	26 (21.7%)
Medicines optimisation in care homes pathway	8 (6.7%)
Primary care pharmacy education pathway	73 (60.8%)
On-the-job training	38 (31.7%)
Other	17 (14.2%)
**Employing organisation**	
PCN	78 (65%)
GP practice	30 (25%)
Other	12 (10%)

GP, general practitioner; PCN, primary care network; SMRs, structured medication reviews.

Just over half of the respondents reported that they were qualified as Independent Prescribers (IPs) (62%, n=74), that is, non-medical healthcare professionals who assess patients with diagnosed and undiagnosed conditions and make decisions about their clinical management, including prescribing. IPs can prescribe autonomously for any condition within their clinical competence, with the exception of some controlled drugs to treat addiction. During SMRs, in partnership with the person and the multidisciplinary team, they may also stop unnecessary medicines being prescribed, make dosage adjustments, deal with referrals and test requests and carry out follow-up care. 61% (73/120) of respondents had completed, or were currently enrolled in, the approved training pathway for SMR competencies—the Primary Care Pharmacy Education Pathway—a mandatory requirement by the NHS.^10^ However, there was considerable variation in their training backgrounds as shown in table 1.

### How were SMRs undertaken?

#### Patient identification, selection and invitations

In most cases (78%, n=93), the clinical pharmacists themselves were identifying eligible patients via electronic health record (EHR) searches based on the nationally identified priority cohorts, or using a GP contract-oriented population health tool (‘Ardens’). PCN clinical leads (38%, n=45), GP partners/practice leads (35%, n=42), and pharmacy technicians (23%, n=27) and occasionally administrative staff were also reported to play a role in identifying eligible patients.

A total of 45/115 (39%) respondents indicated that other patient groups beyond those listed in the primary care contract guidance (care home residents, polypharmacy, patients on medicines commonly associated with medication errors, patients with severe frailty and/or using potentially addictive pain management medication)^10^ were also being identified for SMRs in their organisations (see table 2). These other patient groups included individuals taking potentially problematic medicines prioritised by NHSE in 2022/23,^11^ and individuals referred for reactive SMRs where a particular need for review was identified by other healthcare professionals, patients themselves, carers or family members.

**Table 2:** Patient identification and selection for structured medication reviews

	Respondents n (%)
Who identifies patients for SMRs in the organisations you work for?*	
Primary care network	45 (37.5%)
GP partners or practice lead	42 (35%)
Patients referred by other clinicians	54 (45%)
Pharmacists	93 (77.5%)
Pharmacy technicians	27 (22.5%)
I don’t know	3 (2.5%)
Other†	17 (14.2%)
Which patient groups receive SMRs in the organisations you work for?*	
People in care homes	87 (72.5%)
People on 10 or more medicines	92 (76.7%)
People on medicines are commonly associated with medication errors	99 (82.5%)
People with severe frailty	97 (80.8%)
People using one or more potentially addictive medications, for example, opioids, gabapentinoids, benzodiazepines and z-drugs	94 (78.3%)
Other‡	45 (39.1%)

* 120 respondents were asked to tick all that apply.

† Ardens/EMIS searches, medicines manager, administrative team, care coordinator, patients self-referring.

‡ People with incentivised medicines-related indicators listed by NHS England,^16^ people with frequent hospital admissions, people with mental health problems, people who are housebound, people using medication compliance aids or with compliance issues, people with complex discharge needs, people on 4–9 medicines, people with a clinical need as requested by other health professionals, patients, carers or family members.

GP, general practitioner; SMRs, structured medication reviews.

Invitations to SMRs were most commonly oral or electronic appointment notices, including via phone text messaging (SMS), as reported by 54% (n=76) of respondents. Only 26% (n=36) of respondents reported providing patients with information on the benefits of SMRs or setting expectations for the review process in advance. Sometimes patients were being invited by ‘cold calling’ (see online supplemental Data file).

#### The SMR consultation

When asked who usually conducts SMRs in their organisation, 72% (n=86) of respondents indicated clinical pharmacists, 34% (n=41) indicated GPs, or other healthcare professionals such as Advanced Nurse Practitioners (9%, n=11). When asked about tools/checklists used to identify potentially inappropriate medicines prior to the review, the most common was to calculate Anticholinergic Burden scores (34.6%, n=94), a measure of the combined effect of all medications with anticholinergic activity that a person is taking (see online supplemental Data file). Higher scores have been linked to greater risk of adverse effects such as cognitive impairment, functional decline, falls and increased mortality in older adults.^18^ Perhaps unsurprisingly, due to the number of different patient groups receiving SMRs, aside from checking patients’ medication lists, there was considerable variation in the types of information our respondents reported checking in preparation for/during SMRs (see table 3). The activities reportedly most likely to always be initiated during SMRs were medication adherence checking and identifying potentially inappropriate, problematic or high-risk medicines. The activity least likely to always be initiated was follow-up planning (see table 4).

**Table 3:** Likelihood of information/additional sources being checked in preparation for/during structured medication reviews

	Always	Often	Sometimes	Rarely	Never
Current medication list	120 (100%)	0	0	0	0
Problem list or medical history	111 (92.5%)	8 (6.7%)	0	1 (0.8%)	0
Other investigations and/or test results	93 (77.5%)	22 (18.3%)	4 (3.3%)	1 (0.8%)	0
Kidney function test results	92 (77.3%)	21 (17.6%)	3 (2.5%)	2 (1.7%)	1 (0.8%)
Hospital letters or discharge summaries	80 (66.7%)	33 (27.5%)	6 (5%)	1 (0.8%)	0
Blood pressure	73 (60.8%)	42 (35%)	5 (4.2%)	0	0
Over-the-counter medications	68 (57.1%)	28 (23.5%)	18 (15.1%)	5 (4.2%)	0
Safety issues	65 (55.1%)	28 (27.1%)	16 (13.6%)	7 (5.9%)	2 (1.7%)
Herbal/complementary medicine products	64 (54.7%)	23 (19.7%)	17 (14.5%)	10 (8.5%)	3 (2.6%)
Weight	50 (41.7%)	58 (48.3%)	10 (8.3%)	2 (1.7%)	0
Frailty score	35 (29.7%)	17 (14.4%)	44 (37.3%)	16 (13.6%)	6 (5.1%)
Activities of daily living	29 (25%)	30 (25.9%)	41 (35.3%)	12 (10.3%)	4 (3.4%)
Diet and fluid intake	28 (24.1%)	24 (20.7%)	44 (37.9%)	19 (16.4%)	1 (0.9%)
Falls history	28 (23.7%)	35 (29.7%)	44 (37.3%)	10 (8.5%)	1 (0.8%)
Living circumstances	26 (22.4%)	33 (28.4%)	42 (36.2%)	9 (7.8%)	6 (5.2%)
Capacity assessment	24 (21.1%)	13 (11.4%)	32 (28.1%)	28 (24.6%)	17 (14.9%)
Information from carers or families	22 (18.7%)	32 (27.1%)	53 (44.9%)	10 (8.5%)	1 (0.8%)
Social support	20 (17.5%)	33 (28.9%)	40 (35.1%)	15 (13.2%)	6 (5.3%)
Advance care plan	16 (13.6%)	15 (12.7%)	31 (26.3%)	34 (28.8%)	22 (18.6%)
Continence	16 (13.8%)	19 (16.4%)	53 (45.7%)	24 (20.7%)	4 (3.4%)

**Table 4:** Likelihood of activities being initiated during structured medication reviews

	Always	Often	Sometimes	Rarely	Never
Medicines adherence check	105 (89%)	13 (11%)	0	0	0
Identifying potentially inappropriate, problematic or high-risk medicines	93 (78.8%)	25 (21.2%)	0	0	0
Agreeing on an action plan	90 (76.9%)	26 (22.2%)	1 (0.9%)	0	0
Safety-netting	88 (74.6%)	27 (22.9%)	3 (2.5%)	0	0
Patient’s medical history	81 (68.6%)	28 (23.7%)	8 (6.8%)	1 (68.6%)	0
Medicines reconciliation	74 (63.2%)	32 (27.4%)	8 (6.8%)	2 (1.7%)	1 (0.9%)
Identifying what matters most to patients	72 (60.5%)	37 (31.1%)	6 (5%)	4 (3.4%)	0
Negotiating shared agenda and goals	71 (60.2%)	39 (33.1%)	8 (6.8%)	0	0
Follow-up planning	69 (59%)	34 (29.1%)	14 (12%)	0	0

**Figure 1:**
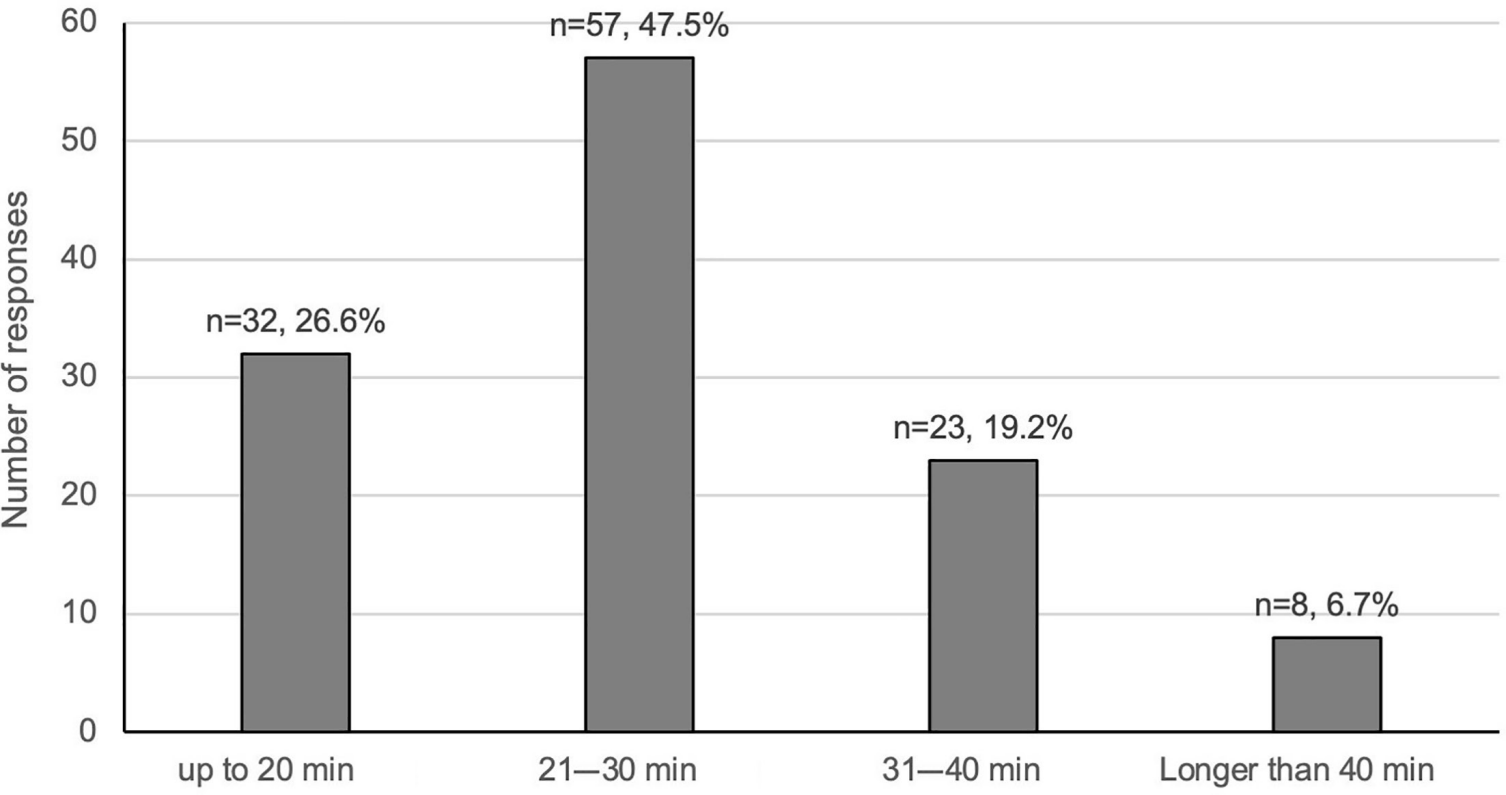
Length of structured medication reviews.

Reviews were nearly always conducted directly with the patient, with only 6.7% (n=8) respondents reporting desk-based reviews. In terms of modality, 98% (n=117) of respondents were conducting telephone SMRs, with 75% (n=90) conducting face-to-face SMRs (see online supplemental Data file). Home visits were also undertaken by 23% (n=27) of respondents. SMR consultation length (not including preparatory or follow-up work) varied considerably, but most reported a length of ‘between 21 and 30 min’ (n=57, 47.5%) (figure 1). Most respondents (n=86) reported using a bespoke SMR template within the patient’s EHR to structure and document the review (see online supplemental Data file).

#### SMRs in care homes

A total of 48% (n=57) of respondents reported undertaking SMRs in care homes. Several respondents commented that these tended to be longer and more complex, requiring more preparatory work and follow-up compared with other settings. Respondents reported often conducting these reviews without direct patient involvement due to mental capacity or communication issues, relying instead on input from care home staff and medication administration records. Two respondents commented that these SMRs were more likely to be geared towards deprescribing where appropriate.

#### Post-SMR consultation

Post-SMR discussion with GPs regarding medicine change interventions differed depending on whether respondents reported being qualified as an IP or not. Non-IPs were more likely than IPs to report seeking advice from a GP when they had concerns about adverse effects or monitoring; when prescribing a new medicine; when recommending changes to already prescribed medicines or when they felt uncertain about an intervention (see table 5).

**Table 5:** The likelihood of discussing medicine change interventions with a general practitioner by an independent prescriber with a status

Independent prescriber (IP) status	IP (n=74)	Non-IP (n=46)	Total* (n=120)
When I have concerns about adverse effects	38 (51.4%)	31 (67.4%)	69 (57.5%)
When I have concerns about monitoring	32 (43.2%)	30 (65.2%)	62 (51.7%)
When I prescribe a new medicine	13 (17.6%)	25 (54.3%)	38 (31.7%)
When I prescribe a dose adjustment	16 (21.6%)	27 (58.7%)	43 (35.8%)
When I recommend stopping a medicine	22 (29.7%)	34 (73.9%)	56 (46.7%)
When I feel uncertain about an intervention	67 (90.5%)	44 (95.7%)	111 (92.5%)
I have never needed to do this	0 (0%)	0 (0%)	0 (0%)
Other†	11 (14.9%)	2 (4.3%)	13 (10.8%)

* Percentage of respondents who selected each answer option.

† When decision is outside of scope of practice, I have concerns I need to discuss with a colleague who may know patient better, to ensure we are on the same page with the patient’s treatment plan, when I feel information needs to be shared, if I have any concerns/need advice regarding the plan, or occasionally at the patient’s request, when specific requests/contributions from other multi-disciplinary team members are required, to notify general practitioner of changes made and any monitoring/review needed.

### Challenges to successful delivery and sustainability of SMRs

Free text responses were provided by 94/120 (78%) of respondents to the question ‘What barriers, if any, have you faced conducting SMRs’. Three main themes were generated from these data: time constraints around the delivery of SMRs, issues relating to organisational infrastructure and wider context and lack of ‘buy-in’ and support from the wider primary care team.

#### Time constraints

Time was the most reported barrier to conducting SMRs, mentioned in 46/94 (49%) of the free text responses. Many respondents expressed that they did not have enough time allocated to prepare for, conduct and/or document SMRs, particularly when conducting reviews with patients who were complex and/or had complex polypharmacy. Time as a barrier was reported by respondents regardless of IP status. Communication difficulties in patients, such as poor hearing for older patients or conducting SMRs in care homes, could also necessitate more time:

30 mins is not long enough to do a thorough review, especially those in care homes, frailty, polypharmacy, etc (R95)

Respondents advocated for a flexible approach with more or less time allocated depending on the complexity. Although there was a lack of agreement about how much time was needed for an SMR, there was evidence that some respondents were frustrated if given 15 mins or less.

Time. Almost all surgeries I work for expect SMRs to be done within 15 min, no matter how many meds pt on or how complex the medication regimen is. The surgery don’t seem to take into account that some patients are elderly and need time to collect their thoughts and speak. Also, a lot of patients have a lot to say about their medications and 15 min is simply not enough time to do an SMR on patient on more than 10 meds (R8)

Several respondents reported minimisation of their concerns regarding lack of time, with one recounting that they resorted to threatening to leave to negotiate more time to manage the workload. Others felt pressure to conduct a certain number of SMRs. However, the average time allocated for SMRs appeared to vary from practice to practice.

I have 45 min for a face-to-face SMR, which allows time for a holistic approach to shared decision making and an effective consultation (R80)

#### Organisational and wider context

The responses suggested that in some organisations, the way that SMRs were being operationalised, for example, without administrative support and insufficient physical space, was impeding effective delivery. This then increased the workload and hence the time required to undertake individual SMRs. For example, some respondents reported having no administrative support available to manage appointment-booking or to be alerted to patients’ mobility or sensory needs, and a shortage of consultation rooms in which to conduct face-to-face reviews. In particular, the mode of consultation made available to clinical pharmacists was not always felt to be suitable for patients:

The current default is to conduct reviews over the phone (R52)

Some respondents felt that more information could be shared in a face-to-face review as opposed to over the telephone. Although housebound patients would greatly benefit from home visits, this opportunity was often not available:

We are currently not doing home visits (R50)

Moreover, the preparatory work needed to support comprehensive SMRs, such as up-to-date test results or other clinical investigations, was often not done and as such, a barrier to efficiency:

Getting patients booked in at the right time, that is, with recent bloods and not duplicating other long-term condition reviews (R49)

Beyond individual organisations, wider issues also contributed to barriers to SMR delivery, such as changing targets and priorities. For example, combining NHSE’s priority patient groups with additional incentivised targets meant that searches often identified too many patients, or failed to pick up higher-risk patients who might benefit the most from a holistic review of all their medicines. These targets were often seen as burdensome and necessitated the completion of a certain number of SMRs by the end of the financial year, putting time stress on clinical pharmacists. Additionally, some respondents found that national priorities for other types of reviews, such as long-term conditions review, meant SMRs were sidelined. Moreover, several respondents mentioned that patients wanted to talk about acute issues, not medication, due to a misunderstanding of the purpose or relaying struggles with access to other clinicians in the organisation:

Patients having their own agenda and a multitude of problems that need addressing especially as they have finally managed to speak to somebody from the practice! (R19)

Outside of the GP practice, care home staff did not always have time to support clinical pharmacists reviewing patients who lacked mental capacity for meaningful involvement. Finally, one respondent reported the availability of suitable local services for patient referrals as inconsistent, hindering the effectiveness of SMRs.

#### Lack of ‘buy-in’ and support

The perceived lack of time, administrative support and physical space issues reported by respondents were often directly linked by them to a lack of understanding or ‘buy-in’ from the wider primary care team regarding their role and the perceived value of SMRs. For example, one respondent mentioned a ‘generally poor perception of importance of the review’ (R7). Some respondents felt that GPs saw SMRs as a tick-box exercise which undermined the validity of the work they were undertaking:

GPs think it’s a tick box exercise; they do not see it as a holistic review led by the patient. Due to this, GPs are not supportive. Some GPs are not available for debrief. GPs and others question the time it takes to do a full SMR (R12).

Another respondent reported feeling unsupported and isolated:

There is a lot of pressure on pharmacists to do this work alone and with very little support from the team to help with this huge piece of work (R57)

Some respondents felt unsupported as members of the primary care team. Several respondents mentioned a lack of confidence or lack of knowledge of what to do with certain issues that arise from SMRs, or insufficient training, as a barrier to effective SMRs. For example, one respondent noted:

GPs/pharmacists in other practices are working on potentially addictive drugs, but we feel this is a specialist area and requires more training for most pharmacists and even GPs (R11)

Furthermore, they felt that protected time for supervision, where they could discuss their work with senior team members, was not always made available.

Finally, several respondents also reported a lack of ‘buy-in’ from patients themselves as barriers, exemplified by poor uptake of SMR invitations: ‘Patient uptake is only 20% following written invitation explaining benefits’ (R10). Another perceived barrier was patients lacking understanding of the purpose of SMRs and some being resistant to speaking with someone new and expressing a preference ‘to speak to a doctor and not a pharmacist’ (R105), particularly in discussions centred on the reduction of opioid medications. One respondent mentioned a barrier in the form of the inclination of some patients to prioritise pharmacological treatments over lifestyle modifications, which could serve as preventive or adjunctive therapeutic measures.

## Discussion

### Overview of findings

This study has identified a number of challenges for clinical pharmacists delivering SMRs in primary care settings across England. Some respondents reported conducting SMRs prior to national implementation. This may be a reflection of the many different types of medication review described in policy documents over the years,^6^ and perhaps some respondents likening SMRs to other types of pharmacist-led medication reviews.^5^

Patients receiving SMRs included individuals meeting NHSE’s criteria for SMRs^10^ and other priority cohorts incentivised at the time.^11^ Over two-thirds of respondents had completed or were enrolled in training specifically designed to support clinical pharmacists conducting SMRs. However, only 14% (8/57) of respondents involved in care home work had completed or were enrolled in medicines optimisation in care homes training. SMRs were being conducted remotely or face-to-face in GP surgeries, care homes and in peoples’ own homes, with telephone SMRs as the most frequently used modality. The majority of reviews were undertaken within a 21–30 min time slot.

A number of important barriers to the successful implementation of SMRs were highlighted. Time constraints were the most reported barrier, especially where there was an expectation from employers or managers that SMR could be done in 15 min or less. There was also evidence of a mismatch between some of our respondents’ perceptions of the value of SMRs—a complex intervention with complex patients which required multiple different tasks that could not be simplified into one short consultation—with perceptions of other practice staff (most commonly GPs). A lack of ‘buy-in’ from patients was also reported in the form of low uptake. This may be related to a lack of information provision, resulting in patient uncertainty as to the offer and a lack of understanding of the role of clinical pharmacists working in primary care.

### Comparisons to existing literature

The importance of having sufficient time for the independent review of prescribing in primary care, in particular with more complex patients (where complexity can include medical and non-medical factors),^19^ was anticipated in the original guidance: ‘We expect that an SMR would take considerably longer than an average GP appointment, although the exact length should vary in line with the needs of the individual patients. Employers should allow for flexibility in appointment length for SMRs, depending on the complexity of individual cases’.^8^ Clinical pharmacists are often tasked with managing complex patients presenting with intricate medication regimens and multiple comorbidities, necessitating an exhaustive review process that extends beyond the standard consultation duration.^9^ Time was also reported as a key theme in a prior qualitative interview study capturing early implementation of telephone SMRs (between September 2020 and February 2022).^13^ Our respondents reported that SMRs required additional time and presented greater challenges, for example, compared with the now discontinued ‘Medicines Use Review’ service in community pharmacies.^20^

A supportive organisational context is important for the successful implementation of any new intervention. Prior research has documented that supportive working relationships between clinical pharmacists and GPs are key to successful integration of the clinical pharmacy workforce in general practice.^21^ Also, a lack of ‘buy-in’ from GPs and patients reinforces pharmacists’ feelings of professional isolation and uncertainty regarding their autonomous role in patient care.^14^ Some of our respondents’ concerns around ‘buy-in’ from wider primary care team members might suggest unclear or differing expectations around the purpose of SMRs.

As argued by Blenkinsopp *et al*,^22^ clinical medication reviews, such as SMRs also provide opportunities for ‘educational intervention to support patient knowledge and adherence’. However, this requires acceptance of the need for review and trust in the reviewer. Patients often prefer to speak to a doctor instead of a pharmacist, which indicates a need for more education about the role of clinical pharmacists in SMRs.^22^ Our respondents’ reports of a lack of uptake from patients may reflect confusion over the clinical pharmacists’ role in general practice, leading to reduced confidence in the SMR process.^23^

### Strengths and weaknesses

This study documents some of the real-world challenges being faced by clinical pharmacists implementing SMRs in primary care in England. Despite this, it only represents a ‘snap-shot’ of 120 clinical pharmacists’ experiences at a specific point in time. Patients and GPs, for example, may have had a different view. The survey may also have been subject to respondent bias by relying on voluntary participation with no reimbursement, and not reaching clinical pharmacists who were not on email distribution lists or active users of social media channels. Additionally, the dependence on self-reported information could lead to recall bias, and the absence of a longitudinal design restricts the capacity to evaluate changes over time. Future research should explore the use of mixed-method approaches to cross-verify data from different sources and offer a more thorough insight into SMR implementation. Finally, SMRs are being undertaken within an ever-changing policy context; for example, halfway through our data collection period, additional financial incentives for SMRs were unexpectedly retired.

### Implications for practice, policy and research

In 2021, NHSE guidance recommended that the appointment length for SMRs should be around 30 min.^10^ Our survey findings highlight that this is not happening uniformly in practice, and in some cases, 30 min was not deemed to be enough time. To overcome these challenges, employers could introduce structured time management policies and establish support systems, including further training and clinical supervision. Local clinical leaders might take note that our findings suggest many clinical pharmacists find it extremely challenging to deliver SMRs in less time than required. Individuals responsible for time/resource allocation should ensure consideration of the SMR workflow in its entirety: patient selection and preparation, appointment booking, review of patients’ EHR, SMR delivery and documentation and where necessary, consulting with GPs and completing any follow-up actions. Furthermore, the complex nature of some patients within the priority cohorts targeted for SMRs may be out of scope in terms of their knowledge and skills. This could have implications for the quality and safety of patient care. In such instances, the most appropriate healthcare professional to carry out such a review should be determined locally.

Where patient uptake is low, this could be remedied by better information provision about the purpose of SMRs that reaches and is understood by people from different ethnic and social backgrounds. More recently, the Health Innovation Network has produced a number of resources which can be used by GP surgeries to aid promotion and patient education about SMRs.^24^ Although this study has allowed insight into some of the barriers to SMR implementation, a more in-depth understanding of the nature of work required from clinical pharmacists, other members of the primary care team and patients (or their caregivers) themselves, not only to undertake SMRs but to implement any changes, is warranted. Our future research will address this through a series of in-depth practice case studies, including patient interviews.

Our own research, and that of others, suggests that wider policy decisions surrounding SMRs may have made implementation more difficult. For example, many of our respondents reported populations receiving SMRs closely aligned with incentivised targets, but some had found that this identified too many patients to review. The retirement of these financial incentives in April 2023, plus the reported lack of ‘buy-in’ from the wider primary care team, has meant that some practices have deprioritised SMRs.^25^ However, NHS statistics show that SMR appointments have continued to increase with 3.1 million recorded between April 2023–March 2024.^26^ Policymakers should await the outcome of further research on the effectiveness of SMRs before considering next steps.

## Conclusion

These results offer insights into the new primary care pharmacy workforce and their role in a country-wide initiative to improve the quality and safety of care for patients taking multiple medicines. More clarity is needed over which patient groups should be receiving SMRs, and patients clearly need better preparation to be able to fully engage in SMRs. Building trust between pharmacists, patients and primary care teams, and continuing professional development, support and oversight for clinical pharmacists conducting SMRs, could lead to more efficient medication reviews. However, a formal evaluation of the effectiveness of SMRs to optimise safe medicines use for patients in England is now warranted.

## Supplementary material

10.1136/bmjopen-2024-097012 online supplemental file 1

10.1136/bmjopen-2024-097012 online supplemental file 2

## Data Availability

Data are available upon reasonable request.
