# Supplementary material for: Understanding structured medication reviews delivered by clinical pharmacists in primary care in England: a national cross-sectional survey
Source: BMJ Open. 2025 Sep 30;15(9):e097012. doi: 10.1136/bmjopen-2024-097012 (PMC12496104; doi:10.1136/bmjopen-2024-097012)
Supplement: online supplemental file 1 [file bmjopen-15-9-s001.pdf]

OSCAR Survey for Pharmacists (Final approved)

Introduction to the OSCAR Study

The NHS aims to reduce medicines-related harm through pharmacist-led structured medication reviews. The OSCAR study aims to understand how structured medication reviews for patients with multiple long-term conditions are being conducted in England, what the challenges are, and how to best optimise them in the future. OSCAR is a National Applied Research Collaboration (ARC) project funded by the National Institute for Health Research (NIHR), and sponsored by the University of Oxford. As part of this study, we are conducting a survey of pharmacists involved in these reviews about their role in implementation. The survey is largely made up of multiple choice questions and should take no more than twenty minutes to complete. There is also an option to save your responses and complete them later. By filling out the survey you are giving your consent for your responses to be used in our study. All responses will be anonymous. We are also recruiting participants for qualitative case studies and interviews. For more information, please leave your name and contact details at the end of the survey. This study has been reviewed and given a favourable opinion by South Central - Hampshire A Research Ethics Committee (22/SC/0373). Thank you for helping us with our research. Professor Richard McManus (Chief Investigator), NIHR ARC Oxford and Thames Valley Photo by Towfiqu barbhuiya on Unsplash

Eligibility

1. Are you a pharmacist conducting structured medication reviews (SMRs) with primary care patients in England?

- ◯ Yes
- ◯ No

About You

2. Which area/s of the country do you work in?

- [ ] East of England
- [ ] East Midlands
- [ ] Greater Manchester
- [ ] Kent, Surrey and Sussex
- [ ] North East and North Cumbria
- [ ] North Thames
- [ ] Northwest London
- [ ] North West Coast
- [ ] Oxford and Thames Valley
- [ ] South London
- [ ] South West Peninsula
- [ ] Wessex
- [ ] West
- [ ] West Midlands
- [ ] Yorkshire and Humber

3. What type of organisation are you employed by?

- ○ PCN
- ○ GP Practice
- ○ Other

4. If you selected Other, please specify:

5. How many GP surgeries do you usually conduct SMRs for?

- ○ 1
- ○ 2

◯ 3 or more

6. What is the nature of your employment?

◯ Full-time
◯ Part-time

7. Are you a remote worker?

◯ Yes
◯ No

8. What is the nature of your employment contract?

◯ Permanent
◯ Fixed-term
◯ Other

9. If you selected Other, please specify:

10. How long have you been conducting SMRs?

◯ 0-5 months
◯ 6-11 months
◯ 1-2 years
◯ 3-5 years
◯ 6 years or more

11. Are you an independent prescriber?

◯ Yes
◯ No

12. Have you completed or enrolled on any of the following training courses? (select all that apply)

- [ ] a. Pharmacist in general practice pathway
- [ ] b. Medicines optimisation in care homes pathway
- [ ] c. Primary care pharmacy education pathway
- [ ] d. On the job training
- [ ] Other

13. If you selected Other, please specify:

Identifying patients for Structured Medication Reviews

If you work across more than one organisation, choose the one you conduct most SMRs for to base your answers on.

14. Which of the following patient groups are currently receiving SMRs in the organisation/s you work for? (select all that apply)

- [ ] People in care homes
- [ ] People on 10 or more medicines
- [ ] People on medicines commonly associated with medication errors
- [ ] People with severe frailty
- [ ] People using one or more potentially addictive medications e.g. opioids, gabapentinoids, benzodiazepines and z-drugs
- [ ] I don't know

15. Are other patient groups than those listed above receiving SMRs in the organisations/s you work for?

- ○ Yes
- ○ No

16. If you selected Yes please specify whom and why:

17. Who identifies patients for SMRs in the organisation/s you work for? (select all that apply)

- [ ] Primary Care Network i.e. Clinical leads or board
- [ ] GP Partners or Practice Lead
- [ ] Patients referred by other clinicians
- [ ] Pharmacists
- [ ] Pharmacy technicians

- [ ] I don't know
- [ ] Other

18. If you selected Other, please specify:

Conducting Structured Medication Reviews

19. Who usually conducts SMRs in the organisation/s you work for?

- ☐ Pharmacists only
- ☐ Pharmacists and GPs
- ☐ Other (e.g., ANPs, practice nurses, paramedics)
- ☐ I don't know

20. If you selected Other, please specify:

21. Approximately how many SMRs are conducted in the organisation/s you work for in a typical week?

- ○ 0-5
- ○ 6-10
- ○ 11-15
- ○ 16-20
- ○ More than 20
- ○ I don't know

22. In the organisation/s you work for, how are patients usually invited for their SMRs?

- ☐ Standard invitation only e.g. verbal invitation, letter or SMS
- ☐ Invitation plus advice on the benefits and/or what to expect
- ☐ Other
- ☐ I don't know

23. If you selected Other, please specify:

24. How are SMRs conducted at the organisation/s you work for? (select all that apply)

- [ ] Face-to-face consultation at GP surgery
- [ ] Telephone consultation
- [ ] Remote video consultation
- [ ] Home visits
- [ ] Care home visits
- [ ] Desk-based review
- [ ] Other
- [ ] I don't know

If you are a remote worker, please also indicate how others at the organisation you work for carry out SMRs

25. If you selected Other, please specify:

26. How long on average do your SMRs with patients last (not including preparatory or follow-up work)?

- ( ) Up to 20 minutes
- ( ) 21-30 minutes
- ( ) 31 to 40 minutes
- ( ) Longer than 40 minutes

27. How often do patients have a carer present during your SMRs?

- ( ) Always

- ◯ Often
- ◯ Sometimes
- ◯ Rarely
- ◯ Never

28. Do you do/approach anything differently with SMRs for patients in care homes (e.g. time spent, clinical input, consultation style etc.)?

- ◯ Yes
- ◯ No
- ◯ I don't conduct SMRs with this patient group

29. If you selected Yes, please explain further:

30. How often you check the following information sources in preparation for, or during, SMRs?

Problem list or medical history

- ◯ Always
- ◯ Often
- ◯ Sometimes
- ◯ Rarely
- ◯ Never

Current medication list

- ◯ Always
- ◯ Often
- ◯ Sometimes
- ◯ Rarely
- ◯ Never

Kidney function test results

- Always
- Often
- Sometimes
- Rarely
- Never

Other investigations and/or test results

- Always
- Often
- Sometimes
- Rarely
- Never

Hospital letters or discharge summaries

- Always
- Often
- Sometimes
- Rarely
- Never

Frailty score

- Always
- Often
- Sometimes
- Rarely
- Never

Advance care plan

- Always
- Often
- Sometimes
- Rarely
- Never

31. How often do you seek the following additional information in preparation for, or during, SMRs?

Blood pressure

- ○ Always
- ○ Often
- ○ Sometimes
- ○ Rarely
- ○ Never

Weight

- ○ Always
- ○ Often
- ○ Sometimes
- ○ Rarely
- ○ Never

Diet and fluid intake

- ○ Always
- ○ Often
- ○ Sometimes
- ○ Rarely
- ○ Never

Continence

- ○ Always
- ○ Often
- ○ Sometimes
- ○ Rarely
- ○ Never

Falls history

- ○ Always
- ○ Often
- ○ Sometimes
- ○ Rarely
- ○ Never

Living circumstances

- ○ Always
- ○ Often
- ○ Sometimes

- Rarely
- Never

Social support

- Always
- Often
- Sometimes
- Rarely
- Never

Activities of daily living

- Always
- Often
- Sometimes
- Rarely
- Never

Over-the-counter medications

- Always
- Often
- Sometimes
- Rarely
- Never

Herbal/complementary medicine products

- Always
- Often
- Sometimes
- Rarely
- Never

Safety issues

- Always
- Often
- Sometimes
- Rarely
- Never

Information from carers/families

- ◯ Always
- ◯ Often
- ◯ Sometimes
- ◯ Rarely
- ◯ Never

Capacity assessment

- ◯ Always
- ◯ Often
- ◯ Sometimes
- ◯ Rarely
- ◯ Never

32. How often do you initiate the following activities during SMRs?

Identifying what matters most to patients

- ◯ Always
- ◯ Often
- ◯ Sometimes
- ◯ Rarely
- ◯ Never

Negotiating shared agenda and goals

- ◯ Always
- ◯ Often
- ◯ Sometimes
- ◯ Rarely
- ◯ Never

Patient's medical history

- ◯ Always
- ◯ Often
- ◯ Sometimes
- ◯ Rarely
- ◯ Never

Medicines reconciliation

- Always
- Often
- Sometimes
- Rarely
- Never

Identifying potentially inappropriate, problematic or high-risk medicines

- Always
- Often
- Sometimes
- Rarely
- Never

Medicines adherence check

- Always
- Often
- Sometimes
- Rarely
- Never

Agreeing an action plan

- Always
- Often
- Sometimes
- Rarely
- Never

Safety-netting

- Always
- Often
- Sometimes
- Rarely
- Never

Follow-up planning

- Always
- Often
- Sometimes

- Rarely
- Never

33. How do you structure your SMR conversations? (select all that apply)

- [ ] Follow template within Electronic Health record
- [ ] Follow written prompts or checklists outside of Electronic Health record
- [ ] Own individual process
- [ ] Other

34. If you selected Other, please specify:

35. Do you use any of the following tools or checklists routinely during SMRs? (select all that apply)

- [ ] a. Anticholinergic Burden Score
- [ ] b. Frailty Index
- [ ] c. NHS SPS Patient-centred approach
- [ ] d. NHS Scotland Polypharmacy 7 Step process
- [ ] e. 3-Step Iterative Process
- [ ] f. Wales Polypharmacy Guidance
- [ ] g. CONSULT Tool
- [ ] h. STOPPFrail Criteria
- [ ] Other

36. If you selected Other, please specify:

Following your Structured Medication Review

37. How do you routinely document SMRs? (select all that apply)

- [ ] Within a SMR template on the patient's notes
- [ ] Within a different template e.g. for a long term condition, on the patient's notes
- [ ] On the patient's notes, but not within a template
- [ ] On a separate document which is uploaded to the patient's notes
- [ ] Other

38. If you selected Other, please specify:

39. Following your SMR when, if ever, do you discuss interventions with a GP? (select all that apply)

- [ ] When I have concerns about adverse effects
- [ ] When I have concerns about monitoring
- [ ] When I prescribe a new medicine
- [ ] When I prescribe a dose adjustment
- [ ] When I recommend stopping a medicine
- [ ] When I feel uncertain about an intervention
- [ ] I have never needed to do this
- [ ] Other

40. If you selected Other, please specify:

41. What barriers, if any, have you faced conducting SMRs?

Next steps

42. We are also recruiting pharmacists conducting SMRs for qualitative case studies, as well as managers/leaders involved in SMRs/medicines optimisation for interview. Would you be willing to receive information about taking part in our qualitative work?

- ◯ Yes
- ◯ No

43. If you selected Yes, please provide your name and e-mail address.

Your contact details will not be linked back to your survey responses, which will remain anonymous.
