# Supplementary material for: Understanding structured medication reviews delivered by clinical pharmacists in primary care in England: a national cross-sectional survey
Source: BMJ Open. 2025 Sep 30;15(9):e097012. doi: 10.1136/bmjopen-2024-097012 (PMC12496104; doi:10.1136/bmjopen-2024-097012)
Supplement: online supplemental file 2 [file bmjopen-15-9-s002.pdf]

Supplementary Data File | Additional survey results

In the organisation/s you work for, how are patients usually invited for their SMRs?*

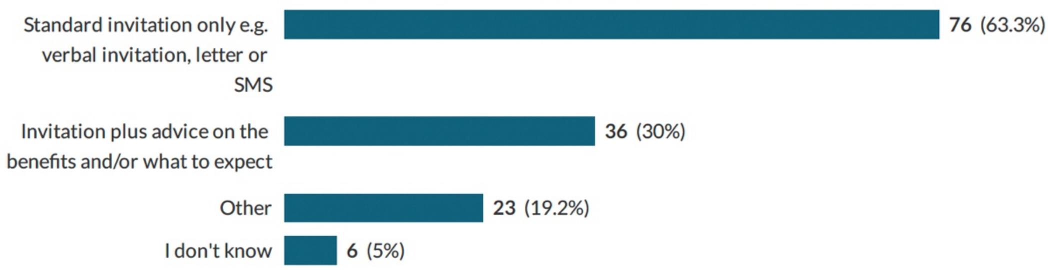

Other: Included ad-hoc if pharmacist/GP identified an issue, not pre booked – cold call or cold call with offer to call back/see, generally conducted as part of their annual medication review

Do you use any of the following tools or checklists routinely during SMRs?*

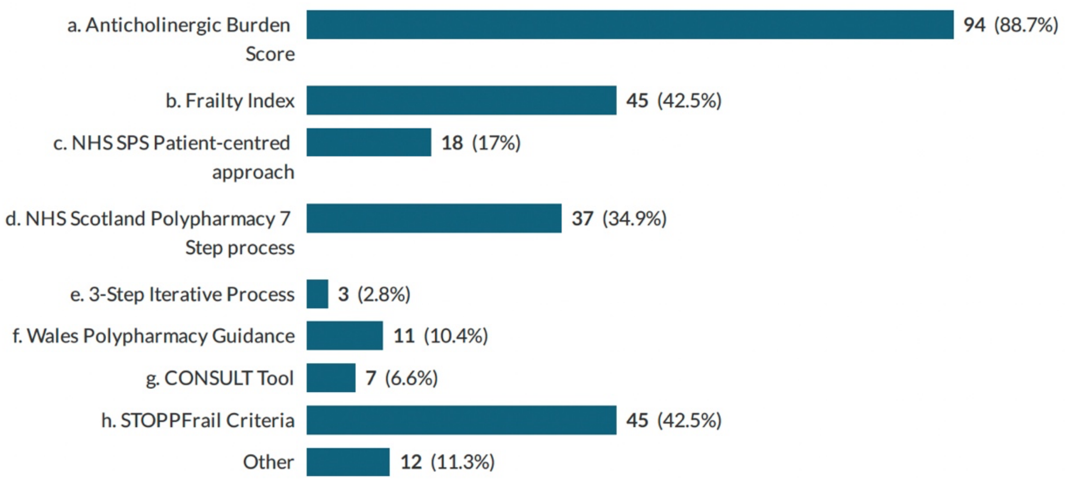

Other: Included not enough time in 20 minutes, NO TEARS, STOPPSTART, OXFORD FALLS, MDCalc for risk scores, Creatinine Clearance, opioid equivalence tool, AEC (Anticholinergic Effect on Cognition) score, SMR live high risk stratification, mix all of the above adapting to patient specifics based on my clinical experience, too many additional things to check with a template open in SystmOne, 30+ years clinical practice, own checklist.

How are SMRs conducted at the organisation/s you work for?*

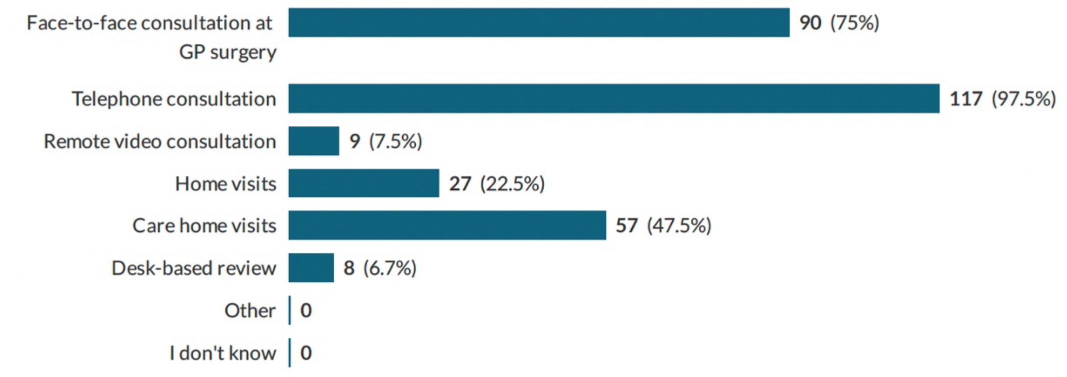

How do you structure your SMR conversations?*

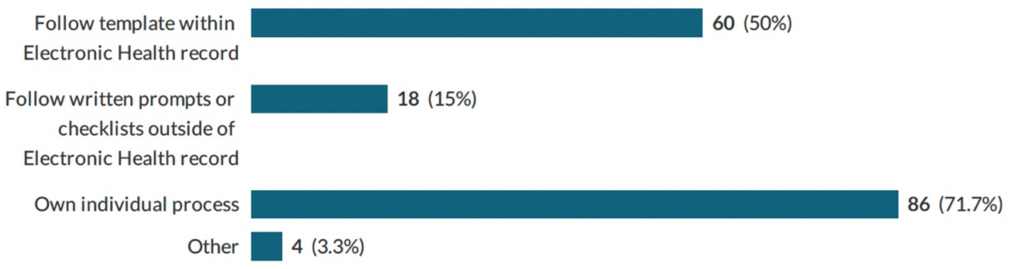

Other: Included explaining my agenda and then letting the patient tell me theirs, a proprietary template, Scottish polypharmacy seven steps, Arden's templates, CCG templates

How do you routinely document SMRs?*

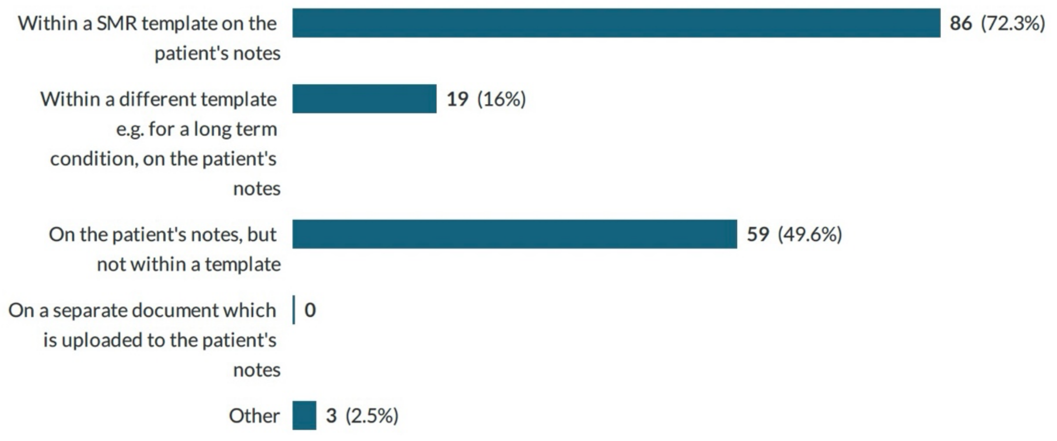

Other: Mix of templates, extra information free typed, a combination of template and free text – some templates do not flow as well or have easy options for recording patient views template.

**Multi answer: Percentage of respondents who selected each answer option*
