# Supplementary material for: Understanding structured medication reviews delivered by clinical pharmacists in primary care in England: a national cross-sectional survey
Source: BMJ Open. 2025 Sep 30;15(9):e097012. doi: 10.1136/bmjopen-2024-097012 (PMC12496104; doi:10.1136/bmjopen-2024-097012)
Supplement: Supplementary file 3 [file bmjopen-2024-097012.reviewer_comments.pdf]

PEER REVIEW HISTORY

BMJ Open publishes all reviews undertaken for accepted manuscripts. Reviewers are asked to complete a checklist review form and are provided with free text boxes to elaborate on their assessment. These free text comments are reproduced below.

ARTICLE DETAILS

Title (Provisional)

Understanding Structured Medication Reviews delivered by clinical pharmacists in primary care in England: a national cross-sectional survey

Authors

Agwunobi, Adaku J; Seeley, Anna E; Tucker, Katherine Louise; Bateman, Paul A; Clark, Christopher Elles; Clegg, Andrew; Ford, Gary; Gadhia, Seema; Hobbs, Richard; Khunti, Kamlesh; Lip, Gregory Y H; de Lusignan, Simon; Mant, Jonathan; McCahon, Deborah; Payne , Rupert A; Perera, Rafael; Seidu, S; Sheppard, James Peter; Williams, Marney; Wright-Drakesmith, Cynthia; McManus, Richard J; Barnes, Rebecca K

VERSION 1 - REVIEW

**Reviewer**	**1**
**Name**	**Sahagún, Ana M.**
**Affiliation**	**Pharmacology, Department of Biomedical Sciences, Faculty of Health Sciences, Institute of Biomedicine (IBIOMED), University of León, 24071 León, Spain**
**Date**	**26-Dec-2024**
**COI**	**None**

I am very grateful for the opportunity to review this manuscript, which I believe is of enormous interest and application. I would also like to make some recommendations that may be of interest to this and its authors.

1.The abstract contains several abbreviations for which no names are given.

2. The conclusions of the abstract are very general.

3. In the Material and Methods section, the data analysis section is very general, and should be described more extensively. There is no indication of how the validation of this novel questionnaire has been carried out. On what basis have the authors designed it? Do you have references of other similar questionnaires? What statistical study have they carried out to validate it and select the questions that make it up?

4. The section “Stakeholder, patient and public involvement” should be repositioned in the Material and Methods section. It is not clear what role it plays in the design and development of the study. It should be organized chronologically.

5. For the open-ended questions, a more specific description should be given of how the groupings of answers have been coded and the members of the research group agreed.

6. There is no section on the statistical study carried out with the data obtained from the questionnaire.

7. More information should be provided on what an independent prescriber (IP) is, especially for the non-British public. What can IPs prescribe? Can they modify medication prescribed by the doctor?

8. The study is too descriptive, without going in depth into the results obtained. With the help of a statistical program, they should obtain a profile of pharmacists using the SMR.

9. Some variables should be grouped together. For example, it does not make much sense to provide descriptive data for 15 regions in England when administratively there are 9. And something similar could be also made with other variables, which may help to develop a statistical study.

10. Conclusions are too general, and should be related to the results obtained in the study.

11. References should be revised according to journal standards.

Reviewer 2

Name Alhindi, Yosra

Affiliation Umm Al-Qura University, clinical pharmacy

Date 01-Jan-2025

COI None

This research is excellent with good work on it

Reviewer 3

Name Moriarty, Frank

Affiliation Royal College of Surgeons in Ireland, HRB Centre for Primary Care Research, Department of General Practice

Date 19-May-2025

COI None

This is a well-written paper that makes an importance and novel contribution to the literature, providing evidence on the real-world implementation of structured medication

reviews in general practice settings by pharmacists in England. The study is well conducted, and will be important in informing further developments in policy on this topic.

1. I have some queries and suggestions for changes to the paper:

Additional clarity on the eligibility criteria for participation would be helpful in the Abstract and Methods. The reference in the Abstract to clinical pharmacists in primary care could potentially be interpreted to include community pharmacists (as I understand the term "clinical pharmacists" can sometimes be ambiguous).

2. In the Abstract and results, problematic drug combinations are referred to as one of the five points in the contract specification, however this is inconsistent with other parts of the text which refers to medicines commonly associated with medication errors.

3. In the Study design section, more detail on the distribution of the weblink would be helpful. Who the "Medicines Optimisation leads and chief pharmacists" are could be described further, and whether this was sent to them for their own participation, or for further distribution (and if so to who). Similarly it would be helpful to describe who the distribution via email channels referred to were directed at.

4. There is a slightly discrepancy between reference to the survey opening on 10th February 2023, and it being conducted between March and May 2023.

5. In the Results, the statement relating to 35% completing SMRs before national implementation could be clarified, to indicate that this is those selecting 3 or more years of experience delivering them. This could also be combined with the previous paragraph which also reports levels of experience.

6. In the Post-SMR consultation section, the results in the table indicate that a higher proportion of non-independent prescribers would seek advice for all points, which is inconsistent with the results text.

7. Would the term non-independent prescribers be appropriate, in the unlikely event some respondents were supplementary prescribers?

8. There are some aspects of the results that are reported in the text but not in tabular format (e.g. care home data, mode of invitation). Was consideration given to reporting all results in the Tables?

9. In the copy of the survey included in the appendix, the average length of reviews question options do not match up with those that are reported in Figure 1. Was an updated version of this used for final data collection?

10. There are several questions included in the survey that have not been reported on, which would provide additional insights to address the study aim. It would be helpful to report on these if they were included in the final data collection survey.

11. Some items in the CROSS checklist could be reported more explicitly in the Methods e.g. approach to pre-testing questionnaire, participant eligibility criteria, if there was any a priori sample size calculation or target.

VERSION 1 - AUTHOR RESPONSE

Reviewer: 1<br>Dr. Ana<br>M. Sahagún, Pharmacology, Department of Biomedical Sciences, Faculty of Health Sciences, Institute of Biomedicine (IBIOMED), University of León, 24071 León, Spain	1.The abstract contains several abbreviations for which no names are given.	Apologies for this oversight. These have now been added.
	2. The conclusions of the abstract are very general.	We have revised the conclusions in the abstract to "These results offer insights into the role being played by the clinical pharmacy workforce in a new country-wide initiative to improve quality and safety of care for patients taking multiple medicines. Better patient preparation and building trust, alongside continuing professional development, more support and oversight for clinical pharmacists conducting SMRs, could lead to more efficient medication reviews. However, formal evaluation of the potential of SMRs to optimise safe medicines use for patients in England is now warranted."

	3. In the Material and Methods section, the data analysis section is very general, and should be described more extensively. There is no indication of how the validation of this novel questionnaire has been carried out. On what basis have the authors designed it? Do you have references of other similar questionnaires? What statistical study have they carried out to validate it and select the questions that make it up?	We wrote "Quantitative data from closed-ended questions were analysed descriptively using functionality within Jisc Online Surveys package" – we have added "(i.e., tools for basic data analysis and reporting, allowing users to visualize, filter and interpret results and to download the results for further analysis)" For the qualitative analysis we have added "NVivo is a software program that is commonly used to support thematic analysis of qualitative data. It allows researchers to organize, code, and analyse data from various sources including surveys, making the process more efficient and systematic". We have also added our response to point 5.<br><br>The design process is described in the 'Development of materials and procedure' subsection. We have added, "We planned to report on the attributes of our respondents (e.g., their employment and prescribing status), their experiences conducting SMRs, and their perceptions (e.g., challenges faced). We were not aware of any existing surveys on SMRs at the time." And "Our approach to data validation included field-testing with pharmacists to seek feedback on question topics, phrasing, formatting, response options, logic and time to complete before and refining and launching the survey."<br><br>Statistical validation studies are more usual for questionnaires (e.g. testing measurement scales) as opposed to surveys (exploring the opinions or

		experiences of a group of people through asking questions).
	4. The section "Stakeholder, patient and public involvement" should be repositioned in the Material and Methods section. It is not clear what role it plays in the design and development of the study. It should be organized chronologically.	The BMJ Open mandates a Patient and Public Involvement statement within the methods section of all submitted manuscripts. We positioned it at the end of the Methods section as our stakeholders and patient representatives played a role throughout the study.
	5. For the open-ended questions, a more specific description should be given of how the groupings of answers have been coded and the members of the research group agreed.	We have added to the Data Analysis subsection "An inductive approach was used to generate themes from the open-ended responses. Four members of the research group met and worked independently through the responses to each of the open-ended questions line-by-line to develop an initial set of codes and categories. These codes and categories were discussed and agreed. The qualitative data were then imported into NVivo and the agreed codes were systematically applied to the data."
	6. There is no section on the statistical study carried out with the data obtained from the questionnaire.	Ours was a survey collecting data on the attributes, experiences and perceptions of primary care pharmacists responding. It was designed to be descriptive rather than a statistical study.
	7. More information should be provided on what an independent prescriber (IP) is, especially for the non-British public.	We described independent prescribers as "non-medical healthcare professionals who assess patients with diagnosed and undiagnosed conditions and make decisions about their clinical management, including prescribing". We have now

	What can IPs prescribe? Can they modify medication prescribed by the doctor?	added to the Participant Characteristics section, “IPs can prescribe autonomously for any condition within their clinical competence, with the exception of some controlled drugs to treat addiction. During SMRs, in partnership with the person and the multidisciplinary team they may also; stop unnecessary medicines being prescribed; make dosage adjustments; deal with referrals and test requests; and carry out follow-up care.”
	8. The study is too descriptive, without going in depth into the results obtained. With the help of a statistical program, they should obtain a profile of pharmacists using the SMR.	As the survey study design was descriptive, further statistical analysis to attempt to explore relationships or make causal inferences would not be appropriate.
	9. Some variables should be grouped together. For example, it does not make much sense to provide descriptive data for 15 regions in England when administratively there are 9. And something similar could be also made with other variables, which may help to develop a statistical study.	The National Institute for Health and Care Research (NIHR) funds 15 Applied Research Collaborations (ARCs) - regional partnerships across England that conduct applied health and care research. As the study was a cross-ARC collaboration, we wanted to try and sample from across as many of these 15 regions as possible. We therefore incorporated these regions as options in one of the survey questions ‘Which area/s of the country do you work in?’ To avoid confusion we have removed reference to the seven regions in NHS England. Please see our response to point 8 above regarding the development of a statistical study.
	10. Conclusions are too general, and should be related to	The conclusion has now been revised to reflect the results obtained.

	the results obtained in the study.	
	11. References should be revised according to journal standards.	The reference formats have now been revised.
Reviewer: 2<br>Dr. Yosra Alhindi, Umm Al-Qura University	This research is excellent with good work on it	Thank you!
Reviewer: 3<br>Dr. Frank Moriarty, Royal College of Surgeons in Ireland	1. Additional clarity on the eligibility criteria for participation would be helpful in the Abstract and Methods. The reference in the Abstract to clinical pharmacists in primary care could potentially be interpreted to include community pharmacists (as I understand the term "clinical pharmacists" can sometimes be ambiguous).	We agree that ‘primary care’ in its widest sense can sometimes include community services such as pharmacies. To avoid further confusion we now only refer to ‘clinical pharmacists’ throughout the manuscript rather than ‘primary care pharmacists’. We have changed the text in the Abstract to, “120 clinical pharmacists with experience of conducting SMRs in primary care”. We have added the following to the Introduction section where it is first mentioned, “The term ‘clinical pharmacist’ is generally understood to mean pharmacists working with patients and other healthcare professionals in a healthcare setting, like a hospital or GP practice, rather than community pharmacy setting.”
	2. In the Abstract and results, problematic drug combinations are referred to as one of the five points in the contract specification, however this is inconsistent with other parts of the text which refers to medicines commonly	Thank you for pointing this out, the use of the term ‘problematic drug combinations’ has now been corrected to ‘medicines commonly associated with medication errors’ where we are referring to the PCN requirements to identify patients who would benefit from a SMR.

	associated with medication errors.	
	3. In the Study design section, more detail on the distribution of the weblink would be helpful. Who the "Medicines Optimisation leads and chief pharmacists" are could be described further, and whether this was sent to them for their own participation, or for further distribution (and if so to who). Similarly it would be helpful to describe who the distribution via email channels referred to were directed at.	Medicines Optimisation leads and Chief Pharmacists usually work at the locality level providing medicines leadership for Integrated Care Boards (42 in England). The idea was that once they received the information they would forward the survey description and weblink out to their clinical pharmacy networks. We are unable to quantify how many individuals the weblink was shared with for further distribution as this was usually done by third parties along the lines of a "snowball effect". As the survey was anonymous we do not know whether those individuals themselves participated in the survey or not. As the study was a cross National Institute for Health and Care Research (NIHR) Applied Research Collaboration (ARC) collaboration, our requests for distribution were sent to communication officers working in the 15 ARCs - regional partnerships across England that conduct applied health and care research. They were also sent to the 15 regional Academic Health Science Networks (AHSNs), established to drive health innovation in the NHS. We asked both types of organisation to send out the weblink via their Twitter accounts and email channels such as primary care newsletters.<br>The study design section has now been revised to include more detail on our distribution strategy.
	4. There is a slightly discrepancy	Thank you for pointing this out, we have now corrected the text to "This was a cross-sectional

	between reference to the survey opening on 10th February 2023, and it being conducted between March and May 2023.	mixed methods survey conducted between February-May 2023"
	5. In the Results, the statement relating to 35% completing SMRs before national implementation could be clarified, to indicate that this is those selecting 3 or more years of experience delivering them. This could also be combined with the previous paragraph which also reports levels of experience.	We have clarified that it was only those selecting six or more years' experience conducting SMRs, despite them only being introduced three years prior, which was particularly notable. We have combined this with the previous paragraph. Thank you for this suggestion.
	6. In the Post-SMR consultation section, the results in the table indicate that a higher proportion of non-independent prescribers would seek advice for all points, which is inconsistent with the results text.	The results reported are consistent with the table which indicates responses filtered by IP vs non-IP status. A key has been added to the table to clarify this.
	7. Would the term non-independent prescribers be appropriate, in the unlikely event some respondents were supplementary prescribers?	Thank you for this suggestion, we have adopted this now.
	8. There are some aspects of the results that are	As a team we discussed which tables and illustrations to prioritise for inclusion, bearing

	reported in the text but not in tabular format (e.g. care home data, mode of invitation). Was consideration given to reporting all results in the Tables?	the guidelines for authors in mind.
	9. In the copy of the survey included in the appendix, the average length of reviews question options do not match up with those that are reported in Figure 1. Was an updated version of this used for final data collection?	Thank you for pointing this out, we have uploaded the final approved pdf version of the survey now.
	10. There are several questions included in the survey that have not been reported on, which would provide additional insights to address the study aim. It would be helpful to report on these if they were included in the final data collection survey.	In ‘The SMR consultation’ subsection, we have now added the results from two further questions in the text, “Most respondents (51.5%, n=86), reported using a bespoke SMR template within the patients EHR to document the review”. And, “When asked about tools/checklists used prior to the review, most respondents reported using Anticholinergic Burden Score (34.6%, n=94) - a measure of the combined effect of all medications with anticholinergic activity that a person is taking. High scores have been linked to greater risk of adverse effects such as cognitive impairment, functional decline, falls, and increased mortality in older adults”. To be fully transparent, in the same subsection we have also reported the results from the three remaining substantive questions with supporting data to be found in Table 3:

		'Likelihood of information/additional sources being checked in preparation for/ during SMRs' and Table 4: 'Likelihood of activities being initiated during SMRs'.
	11. Some items in the CROSS checklist could be reported more explicitly in the Methods e.g. approach to pre-testing questionnaire, participant eligibility criteria, if there was any a priori sample size calculation or target.	Eligibility criteria for participation were pharmacists with experience of conducting SMRs with primary care patients in England. The original target was 30-50 respondents. This information has now been added to the 'Study Design' subsection. We have added further information on field-testing the survey to the 'Development of materials and procedure' subsection as set out in our response to Reviewer 1, point 3. We have also added here "The second page contained an eligibility screening question".

VERSION 2 - REVIEW

Reviewer 3

Name Moriarty, Frank

Affiliation Royal College of Surgeons in Ireland, HRB Centre for Primary Care Research, Department of General Practice

Date 04-Aug-2025

COI

Thank you to the authors for their thorough responses and revisions to the manuscript. I have only two minor comments remaining.

1. I appreciate that there is a large amount of information that has been collected and the consideration the authors have given to presenting selected results in the main text tables. For some aspects, only the most common response is reported in the text (like mode of invitation, tools/checklists used), where full results may be of interest and value to some readers. I would recommend reporting the full results for these questions in supplementary materials to accompany the main reporting in the text.

2. Regarding differences between respondents with IP and non-IP status, my reading of table 4 is that the percentage of non-IP respondents that report discussing issues with a GP is higher than the percentage of IP in all cases. However the Results text does not reflect this.

VERSION 2 - AUTHOR RESPONSE

Reviewer: 3<br>Dr. Frank Moriarty, Royal College of Surgeons in Ireland	1. I appreciate that there is a large amount of information that has been collected and the consideration the authors have given to presenting selected results in the main text tables. For some aspects, only the most common response is reported in the text (like mode of invitation, tools/checklists used), where full results may be of interest and value to some readers. I would recommend reporting the full results for these questions in supplementary materials to accompany the main reporting in the text.	Thank you for this suggestion. We have now added a Supplementary file containing the full results for questions where the results are only partially reported in the Results section. We have also indicated in the text where the reader can find this further information.
	2. Regarding differences between respondents with IP and non-IP status, my reading of table 4 is that the percentage of non-IP respondents that report discussing issues with a GP is higher than the percentage of IP in all cases. However the	Thank you for pointing this out and apologies for this oversight which has now been corrected. We have also made a few small adjustments to the Table legends for clarity and added the 'Other' responses. The text in the Results section now reads: "Non-IPs were more likely than IPs to report seeking advice from a GP when they had concerns about adverse effects or monitoring; when prescribing a new medicine;

	Results text does not reflect this.	when recommending changes to already prescribed medicines; or when they felt uncertain about an intervention (see Table 5)."
